# Analysis of lncRNA and mRNA Repertoires in Lung From BAFF-R-Deficient *Pneumocystis*-Infected Mice

**DOI:** 10.3389/fimmu.2022.898660

**Published:** 2022-06-10

**Authors:** Heng-Mo Rong, Chao Zhang, Guang-Sheng Rong, Ting Li, Xiao-Jun Qian, Dong Wang, Zhao-Hui Tong

**Affiliations:** ^1^Department of Respiratory and Critical Care Medicine, Beijing Institute of Respiratory Medicine and Beijing Chao-Yang Hospital, Capital Medical University, Beijing, China; ^2^Department of Respiratory Medicine, The Third People’s Hospital of Hefei, Hefei, China

**Keywords:** *Pneumocystis* pneumonia, BAFF-R, lncRNA, B cells, immunoregulation

## Abstract

**Background:**

*Pneumocystis* pneumonia (PCP) is a common medical issue in immunosuppressive patients. Increasing evidence supports that B cells may play an essential role in PCP individuals. The present study aims to integrate lncRNA and mRNA expression profiles and further investigate the molecular function of mature B cells in PCP.

**Methods:**

The lung tissue of wild-type (WT) mice and B-cell-activating factor receptor–deficient (mature B-cell deficiency, BAFF-R^–/–^) mice were harvested at 3 weeks after being infected with pneumocystis. After total RNAs were extracted, transcriptome profiling was performed following the Illumina HiSeq 3000 protocol. lncRNA-targeted miRNA pairs were predicted using the online databases. The Kyoto Encyclopedia of Genes and Genomes (KEGG) and Gene Ontology (GO) enrichment pathways were analyzed to functionally annotate these differentially expressed genes. Additionally, the immune-related lncRNA–miRNA–mRNA–ceRNA network was subsequently performed. The quantitative real-time PCR (RT-PCR) analysis was conducted to evaluate the lncRNA and mRNA expression profiles in WT-PCP mice and BAFF-R^–/–^ PCP mice.

**Results:**

Compared with the control group, 166 mRNAs were observed to be aberrantly expressed (fold change value ≥2; *P <*0.05) in the BAFF-R^–/–^ PCP group, including 39 upregulated and 127 downregulated genes, while there were 69 lncRNAs differently expressed in the BAFF-R^–/–^ PCP group, including 15 upregulated and 54 downregulated genes. In addition, GO and KEGG pathway analyses showed that BAFF-R deficiency played an important role in the primary and adaptive immune responses in PCP. Furthermore, the lncRNA and mRNA co-expression network was established. We noted that the core network of lncRNA-TF (transcription factor) pairs could be classified into the categories including infection and immunity pathways.

**Conclusion:**

In summary, in this study, we further explored the role of mature B cells in the pathogenesis and progression of PCP and the data demonstrated that BAFF-R deficiency could play a significant role in immune regulation in the PCP population.

## Introduction

*Pneumocystis* pneumonia (PCP), caused by *Pneumocystis jirovecii*, is a kind of devastating fungal disease in immunocompromised patients. Nowadays, the widespread use of effective antiviral therapies decreased the numbers of HIV-positive patients with PCP, but there is a growing number of HIV-negative PCP individuals who receive immunosuppressive therapies (1). Meanwhile, the non-HIV-infected PCP population is characterized by a more rapid disease progression and higher mortality (2, 3). Thus, the susceptibility of HIV-negative individuals for PCP has drawn the attention of researchers, but the pathogenesis and development of PCP remains to be clarified. Currently, however, the infected mice model remains the main source of laboratory studies and investigators have made improvements in understanding the host–pathogen relationship with *Pneumocystis* in immunology (4). Macrophages play a significant role as the first line of host defense against *Pneumocystis* in the innate immune of pathogen invasion (5, 6). In adaptive immunity, CD4^+^ T cells are the central factor for the clearance of *Pneumocystis* by the recruitment of the other effector cells (7). Furthermore, there are some researchers who verified that T helper cells including Th1, Th2, Th9, and Th17 cells could have the immune regulatory function in the *Pneumocystis*-infected mice model. Additionally, the role of B cells in PCP has also been reported in a few studies and our previous study has also indicated that IL-10-producing B cells could suppress Th1/Th17-cell immune responses in B-cell-activating factor receptor deficient (BAFF-R–/–) PCP mice. It suggested that B cells play an important role in the regulation of Th cells in response to a *Pneumocystis* infection (8).

Noncoding RNAs (NcRNAs) were characterized as the deficiency of a clear potential to encode proteins or peptides, while they could affect the expression of other genes *via* a variety of mechanisms (9). Many studies over the last decades discovered many classes of ncRNA, including microRNA (miRNA), small nucleolar RNA (snoRNA), long ncRNA (lncRNA), and circular RNA (circRNA). LncRNAs are classified into ncRNA 200 bp and have been demonstrated to play critical roles in the modification of genes and post-gene regulation mechanisms (10). In recent decades, the studies of lncRNA suggest that lncRNAs act crucial and diverse roles in the development and progression of various diseases (11). Meanwhile, the function of lncRNAs in the pathogenesis of PCP has not been clearly clarified yet. Our previous study has verified that in *Pneumocystis*-infected mice, there was a great quantity of differentially expressed genes (DEGs) between WT mice and BAFF-R^–/–^ mice and IL-1bete related genes could play an immune regulatory role in Th1/Th17-cell immune responses (8).

In the present study, RNA sequencing was performed to explore the lncRNA and mRNA expression profiles in the lung tissue of WT PCP mice and BAFF-R^–/–^ PCP mice. Additionally, bioinformation analysis including Gene Ontology (GO) and Kyoto Encyclopedia of Genes and Genomes (KEGG) pathway analyses and lncRNA–mRNA co-expression network construction were conducted to identify the underlying biological functions of differentially expressed RNAs.

## Materials and Methods

### Mice

Female 6- to 8-week-old C57BL/6 mice (Vital River Laboratory Animal Co., Ltd, Beijing, China) and BAFF-R^–/–^ mice (stock no. 007212, the Jackson Laboratory, Bar Harbor, ME, United States) were used in this study. Mice were bred in our animal facility and kept in sterile isolated ventilated cages. All animal experiments were approved by the Capital Medical University Animal Care and Use Committee.

### Infection With *Pneumocystis*

*P. murina* was maintained in CB17 SCID mice as previously described with some modifications (8). Briefly, the mice were intratracheally inoculated with *Pneumocystis* cysts and mice in the control group were administered with 100 μl Polybutylene succinate (PBS). The *Pneumocystis* burden in the right lung lobes was evaluated by real-time PCR (RT-PCR) *via* TaqMan assays (**Supplementary Figure 1**). The mice survived at 3 weeks after infection, and we obtained the middle lobe from the right lung of each mouse.

### Transcriptomics Analysis of Lung Tissue

Transcriptome profiling was performed by RiboBio following the Illumina HiSeq 3000 protocol (Guangzhou, China). Firstly, total RNA was extracted from mixed samples of mice using a TRIzol reagent (Invitrogen, Carlsbad, CA, United States). Subsequently, a ribosome kit was used to remove rRNA and then mRNA was purified and fragmented into small pieces with elute, prime, and fragment mixes. After end-repair and adapter-ligation, the products were amplified through PCR and purified to construct the cDNA library. A methodology workflow was presented in the supplementary materials.

The original sequencing data should be filtered, the adaptor removed, and low-quality reads processed to acquire clean data. RPKM (expected number of reads per kilobase of transcript sequence per millions base pairs sequenced) was used to estimate the level of gene expression, which represents the number of reads per million from a gene per kilobase length, considering the effects of sequencing depth and gene length on the read count. The gene expression level of the genome was statistically analyzed to reflect the overall expression level of the sample. Principal component analysis (PCA) is a mathematical algorithm that reduces the dimensions of data while preserving the vast majority of variables in a data set. PCA identifies the principal components to find a direction, and the data distributed along this direction is the maximum, so as to reduce the data dimension. By using such principal components, it is possible to plot the variables related to samples, make the similarities and differences between samples clear, and determine whether different samples can be grouped together.

For the detected mRNA, the gene expression was further calculated and the DEGs among the samples were analyzed. DEGs were analyzed using the DESeq package in R language and the t-test. The differentially expressed mRNAs between the two groups of samples were analyzed with DESeq by negative binomial distribution. After that, DEGs were performed for GO analysis and KEGG pathway analysis. The GO enrichment analysis of DEGs was conducted using the Database for Annotation, Visualization, and Integrated Discovery. The KEGG pathway analysis of differentially expressed mRNAs was conducted to illuminate the cell pathways associated with these genes. The expression of lncRNA was calculated and the differentially expressed lncRNA was analyzed among the samples, and specific annotations were given for the identification of the corresponding Open Reading Frame (ORF), protein domain and coding potential, as well as the prediction of a secondary structure and family analysis.

Furthermore, on the basis of the known gene model of the analyzed species, the new transcription region was predicted by using the sequencing data of all samples, and the expression level of the new transcription region was analyzed. We identified new lncRNAs according to the following strict filtering conditions: (1) filter out known genes and lncRNA in the database; (2) RNAs greater than 200 nt in length; (3) the predicted open dyslexia less than 300 nt; (4) filter out RNAs that have protein domains; (5) filter out potentially coding RNA; and (6) filter out potentially coding RNA. Through the above steps, the transcripts with lncRNA characteristics in the new transcripts were screened out step by step. Finally, those with a coding potential score less than -1 in software were identified as new lncRNA.

### LncRNA Target Gene Prediction

Based on the analysis of differentially expressed lncRNAs and mRNAs, a correlation analysis was conducted on each significantly differentially expressed lncRNA and mRNA. The target genes of lncRNA were predicted by establishing the relationship between lncRNA and mRNA and analyzing the mode of action of lncRNA. LncRNA interacts with target genes in cis and trans methods. lncRNAs could play significant biological roles by regulating the contiguous gene by integrating the differentially expressed lncRNAs of their neighbors (10 KB), thus obtaining potential target genes of lncRNA. Transregulation is the mRNA expression on different chromosomes or the distal end of chromosomes. Blast was used to select the mRNA sequences complementary to lncRNA, and RNAplex was used to calculate the complementary energy between the two sequences to screen out the stable-binding mRNAs. After the interaction between lncRNA and target genes was obtained, the network diagram of the interaction between lncRNA and target genes was drawn.

### Co-Expression Analysis of lncRNAs and mRNAs

We constructed a co-expression network of lncRNAs and protein-coding genes to infer the underlying regulatory function and potential target genes. An expression level matrix of all putative lncRNAs and known lncRNAs was constructed. The most variant protein-coding genes among the 15 samples were included, and the median absolute deviation was used as a variability measurement. The Pearson correlation of each gene pair was calculated using R package WGCNA (weighted gene co-expression network analysis).

### PCR Validation

RNAs were purified and quantified as previously described. RT-PCR was executed by SYBR PreMix Ex Taq TM II ROX (Takara, Dalian, China) and the ABI Prism 7500 Sequence Detection System (Applied Biosystems, Foster City, CA, United States). Gene expression was calculated using the 2^-delta delta CT^ method. GAPDH was used as a reference gene to analyze the gene quantitatively.

### Statistical Analysis

The data of this study were expressed as mean ± SE. The comparison of continuous data was performed by an independent Student’s *t*-test. The Prism 8 (GraphPad Software, San Diego, CA, United States) was used for statistical analysis. *P*-values <0.05 were considered statistically significant. Sequencing data were analyzed by using FC and Student’s t-test. FC ≥2.0 and *P* ≤0.05 were set as the criterion to identify the differentially expressed mRNAs and lncRNAs.

## Results

### Identification of Differentially Expressed lncRNAs and mRNAs

A total of 15 samples from the lung tissue of C57/BL6 mice were used in this study. There were 5 samples from the negative control group, 5 samples from the WT PCP group, and 5 samples from the BAFF-R^–/–^ PCP group, respectively. Removing reads with non-canonical letters or low quality, and discarding the sequences shorter than 18 nt, the mean numbers of clean reads were 117,007,330. In total, 27,343 transcripts were detected in the present study, in which 16,344 transcripts were identified as mRNA and 5,328 transcripts as lncRNA. The saturation of all the genes were detected and shown in **Supplementary Figure 2A**. The RPKM density distribution map was used to investigate the gene expression pattern of the samples, and the density distribution map of genes was shown in **Supplementary Figure 2B**. The results of the PCA analysis of the samples were demonstrated in **Supplementary Figure 2C**.

**Figure 1:**
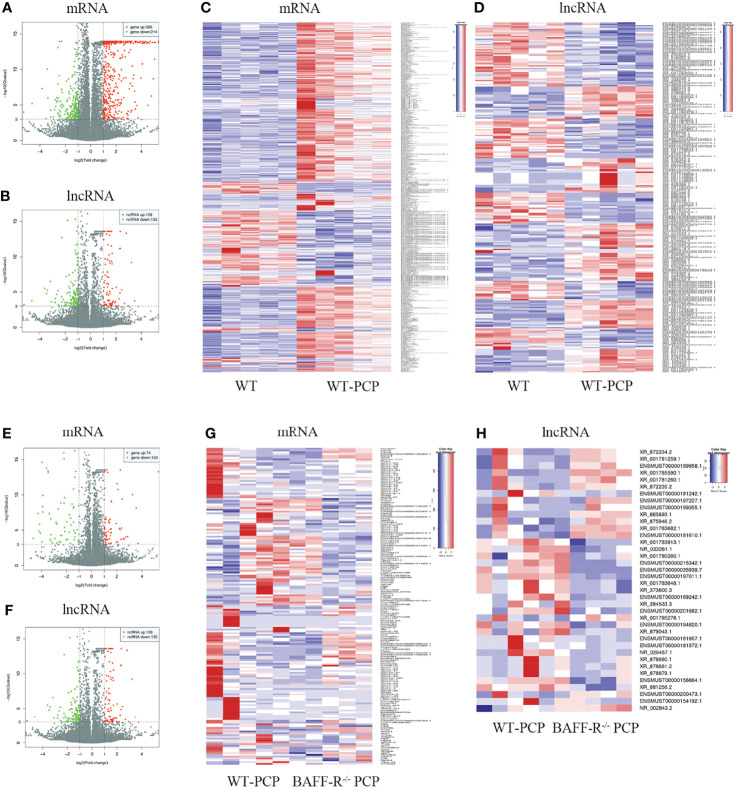
Analysis of differentially expressed genes (DEGs) in the uninfected mice and WT-PCP mice. Volcano plots indicate the respective upregulated and downregulated mRNAs **(A)** and lncRNAs **(B)** in mice from the WT-PCP group compared with those from the control group. A heat map shows the differentially expressed mRNAs **(C)** and lncRNAs **(D)** between the control group and the WT-PCP group. In the clustering analysis, upregulated and downregulated genes are colored in red and purple, respectively. Analysis of DEGs in WT Pneumocystis infected mice and BAFF-R^–/–^ Pneumocystis infected mice. Volcano plots indicate the respective upregulated and downregulated mRNAs **(E)** and lncRNAs **(F)** in mice from the BAFF-R^–/–^ PCP group compared with those from the WT-PCP mice. Heat map shows the differentially expressed mRNAs **(G)** and lncRNAs **(H)** between the WT PCP group and BAFF-R^–/–^ PCP mice. In the clustering analysis, upregulated and downregulated genes are colored in red and purple, respectively.

Using WT uninfected mice as the control group, we analyzed differentially expressed genes between the control group and the WT-PCP group. The top 10 differentially expressed genes (DEGs) are shown in **Table 1**. After removing the low-abundance genes, 501 differentially expressed mRNAs, of which 470 mRNAs were upregulated and 31 mRNAs were downregulated, and 101 differentially expressed lncRNAs, of which 19 lncRNAs were upregulated and 82 lncRNAs were downregulated, were identified between the uninfected mice and WT-PCP mice (**Figures 1A–D**). Compared with the RNA acquired from the lung tissue in WT-PCP mice, we explored 166 mRNAs (39 upregulated and 127 downregulated) and 69 lncRNAs (15 upregulated and 54 downregulated) in BAFF-R^–/–^ PCP mice (**Figures 1E–H**). The top 10 differentially expressed genes are shown in **Table 2**.

**Table 1:** Top 10 differentially expressed lncRNAs and mRNAs between the control group and WT-PCP group.

Top 10 upregulated lncRNA			Top 10 upregulated mRNA		
Gene symbol	LogFC	P-value	Gene symbol	LogFC	P-value
XR_384118.2	2.97	5.28E-05	Chil4	8.80	4.44E-16
XR_875359.1	2.67	2.12E-05	Clca1	7.68	0
NR_027805.1	2.18	5.22E-05	Rnase2a	7.34	2.22E-16
XR_001785252.1	2.01	5.05E-05	Rpl34-ps1	7.18	2.22E-16
XR_377683.2	1.97	5.05E-05	Capn9	6.83	1.11E-15
XR_379717.3	1.69	2.88E-05	Sectm1a	6.11	3.74E-05
XR_881088.2	1.67	4.51E-05	Itln1	6.01	1.21E-10
XR_389045.3	1.52	6.22E-07	Fer1l6	5.73	8.88E-16
XR_389046.3	1.52	6.22E-07	Fbp1	5.66	2.22E-16
XR_880214.2	1.53	6.22E-07	Ubd	5.39	4.44E-16
**Top 10 downregulated lncRNA**			**Top 10 downregulated mRNA**		
XR_872277.1	-5.28	3.67E-05	LOC108168336	-4.62	5.91E-07
XR_872276.2	-5.26	3.67E-05	Upk1a	-3.15	6.79E-05
XR_872278.1	-5.24	3.67E-05	Gm3837	-2.62	2.30E-07
XR_001780316.1	-4.64	7.29E-06	Gm5148	-2.48	4.22E-11
ENSMUST00000181957.1	-4.28	1.91E-20	LOC105244208	-2.46	1.88E-16
ENSMUST00000181242.1	-4.23	0	LOC108167922	-1.87	2.89E-07
XR_374167.3	-3.62	9.66E-08	Nppa	-1.79	3.36E-29
XR_374168.3	-3.48	5.84E-07	Klhl3	-1.61	5.07E-05
ENSMUST00000181572.1	-3.18	6.96E-09	Amy1	-1.39	2.08E-05
NR_040272.1	-2.58	2.15E-07	Foxq1	-1.32	1.94E-12

**Table 2:** Top 10 differentially expressed lncRNAs and mRNAs between WT-PCP and BAFF-R^–/–^ PCP mice.

Top 10 upregulated lncRNAs			Top 10 upregulated mRNAs		
Gene symbol	LogFC	*P*-value	Gene symbol	LogFC	*P*-value
XR_869775.2	5.58	0.002	Nppa	2.49	3.27E-06
ENSMUST00000199065.1	5.33	0.007	Shisa8	2.08	1.93E-07
XR_387599.3	5.14	0.007	Trim10	2.03	2.34E-05
XR_373476.1	5.02	0.014	Havcr1	1.97	3.22E-14
ENSMUST00000187093.1	4.96	0.014	Ngp	1.95	2.34E-05
XR_868133.1	4.92	0.007	Thrsp	1.76	2.97E-08
XR_868134.1	4.92	0.007	Cd163l1	1.58	1.93E-07
XR_871418.1	4.72	0.0002	Stfa2l1	1.57	1.50E-11
XR_868424.1	4.67	0.002	Spta1	1.56	7.65E-07
XR_873200.1	4.66	0.002	Klb	1.55	1.16E-11
**Top 10 downregulated lncRNA**			**Top 10 downregulated mRNA**		
XR_876265.2	-5.40	0.002	Rdm1	-2.28	0
XR_381821.2	-4.51	0.004	Cr2	-1.86	3.25E-25
NR_026942.1	-4.42	0.004	Cacna1i	-1.76	1.28E-09
XR_373980.2	-4.42	0.002	Ocstamp	-1.68	4.04E-05
NR_077222.1	-4.26	0.004	Gm41885	-1.65	5.06E-08
XR_106110.3	-4.04	0.009	Cd19	-1.62	2.60E-26
XR_001780647.1	-4.00	0.001	Pou2af1	-1.59	9.96E-29
XR_874705.2	-3.98	0.02	Fcrl1	-1.55	3.30E-11
XR_001779198.1	-3.92	0.02	Pax5	-1.48	7.04E-20
XR_386758.1	-3.90	0.02	Bank1	-1.38	4.07E-21

**Figure 2:**
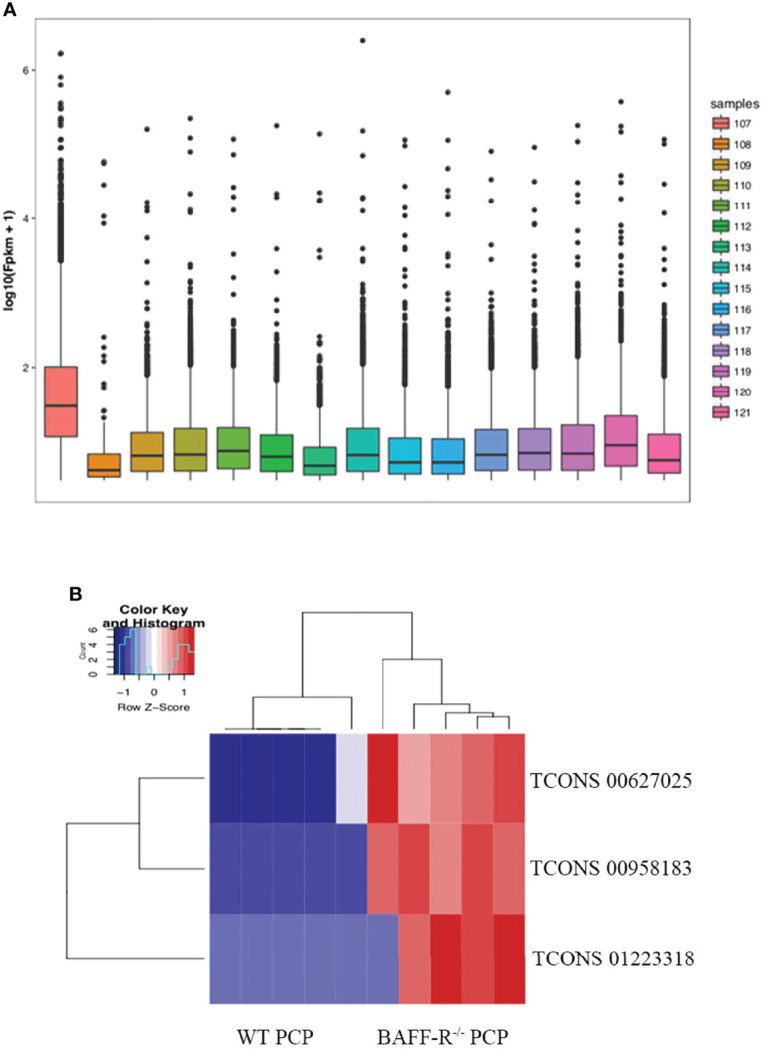
New transcription area and novel lncRNA and in BAFF-R^–/–^ PCP mice. **(A)** Analysis of a new transcription area in the control group, WT PCP group and BAFF-R^–/–^ PCP group. Boxplot uses the minimum, first quartile, median, third quartile, and maximum value of a set of data to reflect the central position and walking range of data distribution, which can roughly judge whether the data are symmetric or not. **(B)** Heat map demonstrates that compared with the WT PCP group, there were 3 novel lncRNAs increased in the BAFF-R^–/–^ PCP group.

Furthermore, the counts of the new transcript genes were detected (**Table 3** and **Figure 2A**) and the results of the differential analysis of new lncRNA expression were shown in **Table 4**. We also found 3 novel lncRNAs increased in BAFF-R^–/–^ PCP mice. On the analysis of the species on the basis of the known genetic model, using all the sample sequence data to forecast the new transcription area (FPKM > 1), the results of the differential analysis of the expression of new lncRNAs were shown in **Figure 2B**.

**Table 3:** The counts of new transcript genes.

Sample Name	New Transcripts Count
107	25,704
108	127
109	7,534
110	7,964
111	8,343
112	6,591
113	2,138
114	5,742
115	3,514
116	3,781
117	7,134
118	8,098
119	4,716
120	6,246
121	4,708

**Table 4:** Differential analysis of new lncRNA expression.

Transcript	Log2 (Fold Change)	p-value	q-value
TCONS 00627025	Inf	3.01551	0.00024
TCONS 00958183	Inf	2.86463	0.00077
TCONS 01223318	7.31646	1.35259	0.00054

### KEGG Pathway and GO Analysis of Differentially Expressed mRNAs

**Figure 3:**
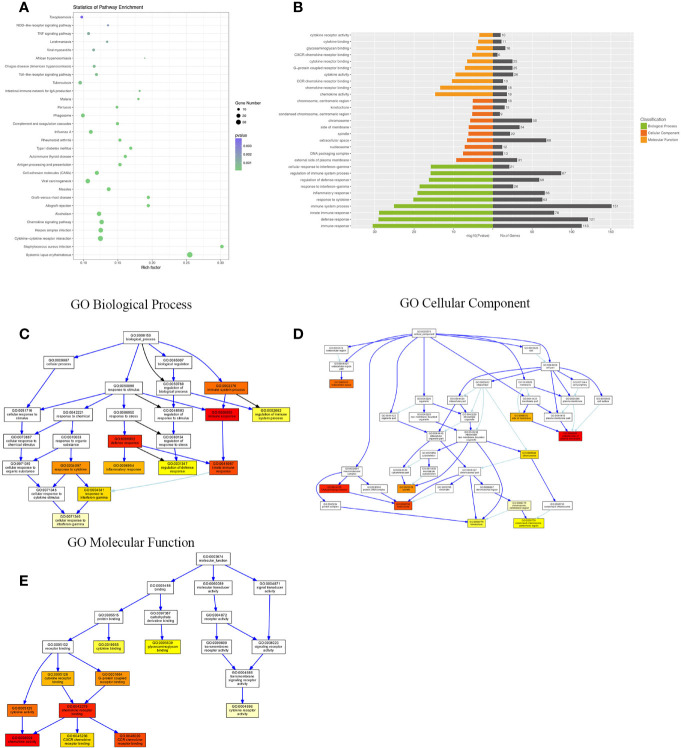
KEGG (Kyoto Encyclopedia of Genes and Genomes) pathway and Gene Ontology (GO) analysis of DEGs between the uninfected mice and the WT-PCP mice. **(A)** KEGG pathway analysis of DEGs between the uninfected mice and the WT-PCP mice. **(B)** GO analysis of DEGs between the uninfected mice and the WT-PCP mice. **(C)** Changes in the biological processes of DEGs. **(D)** Changes in the cellular component of DEGs. **(E)** Changes in the molecular function of DEGs.

Compared with the uninfected mice, KEGG pathway results demonstrated that differentially expressed genes were mainly enriched in the tumor necrosis factor (TNF) signaling pathway, tuberculosis, phagosome, complement and coagulation cascades, viral carcinogenesis, chemokine signaling pathway, cytokine–cytokine receptor interaction, etc. GO analysis data revealed that changes in the biological processes of DEGs were significantly enriched in the innate immune response, inflammatory response, immune system process, plasma membrane, cellular response to interferon-gamma, and regulation of immune system process. Changes in the cellular component of DEGs were mainly enriched in the external side of the plasma membrane, extracellular space, chromosome, and centromeric region. Changes in the molecular function were mostly enriched in the chemokine activity, chemokine receptor binding, CCR chemokine receptor binding, and CXCR chemokine receptor binding (**Figure 3**).

**Figure 4:**
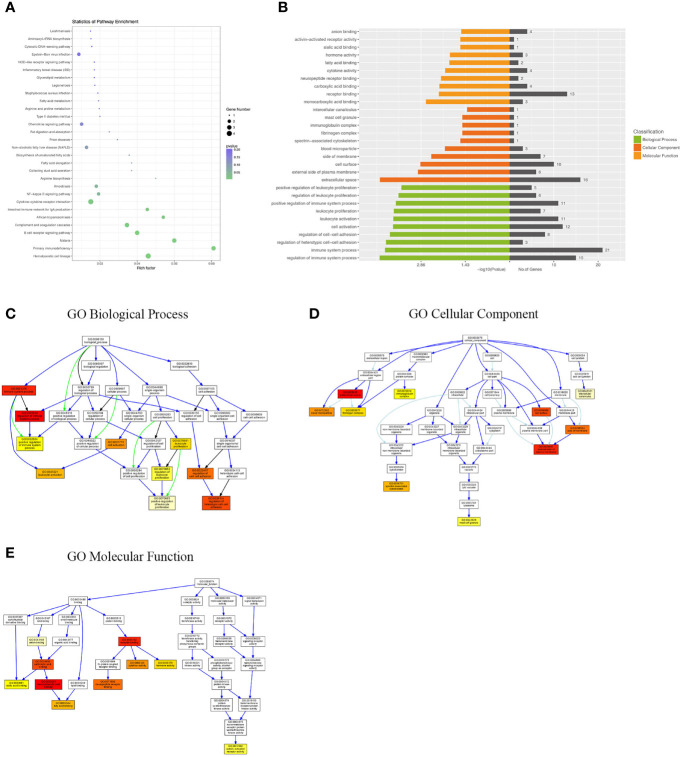
KEGG pathway and GO analysis of DEGs between WT-PCP mice and BAFF-R^–/–^ PCP mice. **(A)** KEGG pathway analysis of DEGs between the WT-PCP group and the BAFF-R^–/–^ PCP group. **(B)** GO analysis of DEGs between the WT-PCP group and the BAFF-R^–/–^ PCP group. **(C)** Changes in the biological processes of DEGs. **(D)** Changes in the cellular component of DEGs. **(E)** Changes in the molecular function of DEGs.

Differentially expressed mRNAs between WT-PCP mice and BAFF-R^–/–^PCP mice were analyzed, and the KEGG pathway results showed that the DEGs were primarily enriched in the Epstein–Barr virus infection, chemokine signaling pathway, non-alcoholic fatty liver disease, NF-kappa B signaling pathway, cytokine–cytokine receptor interaction, complement and coagulation cascades, B-cell receptor signaling pathway, etc (**Figure 4A**). GO analysis results demonstrated that changes in the biological processes of DEGs were significantly enriched in the positive regulation of leukocyte proliferation, regulation of leukocyte proliferation, positive regulation of immune system process, leukocyte proliferation, leukocyte activation, cell activation, regulation of cell–cell adhesion, regulation of regulation of heterotypic cell–cell adhesion, immune system process, and regulation of immune system process. Changes in the cellular component of DEGs were mainly enriched in intercellular canaliculus, the mast cell granule, immunoglobulin complex, fibrinogen complex, spectrin-associated cytoskeleton, blood microparticle, side of the membrane, cell surface, external side of plasma membrane, and extracellular space. Changes in molecular function were mainly enriched in anion binding, activin-activated receptor activity, sialic acid binding, hormone activity, fatty acid binding, cytokine activity, neuropeptide receptor binding, carboxylic acid binding, receptor binding, and monocarboxylic acid binding (**Figures 4B–E**).

### KEGG Pathway and GO Analysis of Differentially Expressed lncRNA-Targeted mRNA

Since most of the functional lncRNAs are mainly executed in protein-coding target genes, we applied the co-expression patterns of mRNA and lncRNA to further study the biological function of these differentially expressed lncRNAs. Using GO and KEGG pathway analyses, we aimed to identify the speculated functions about the target genes of the differentially expressed lncRNAs between the WT-PCP group and the BAFF-R^–/–^ PCP group.

**Figure 5:**
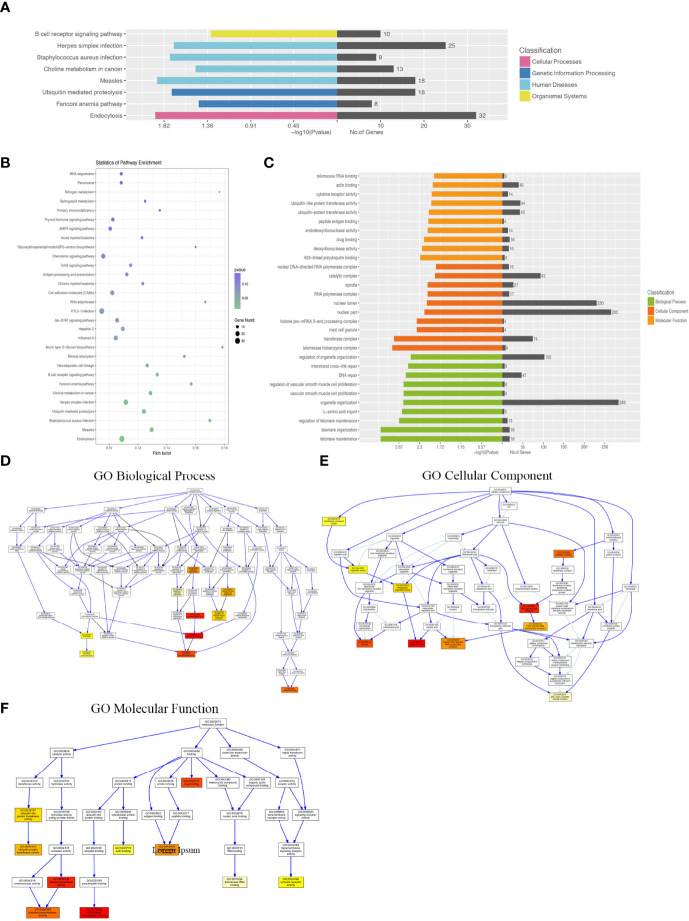
KEGG pathway and GO analysis of differentially expressed lncRNA–target mRNAs between WT-PCP mice and BAFF-R^–/–^ PCP mice. **(A, B)** KEGG pathway analysis of DEGs between WT-PCP mice and BAFF-R^–/–^ PCP mice. **(C)** GO analysis of DEGs between WT-PCP mice and BAFF-R^–/–^ PCP mice. **(D)** Changes in the biological processes of DEGs. **(E)** Changes in the cellular component of DEGs. **(F)** Changes in the molecular function of DEGs.

KEGG pathway analysis demonstrated that the DEGs were mainly enriched in the B-cell receptor signaling pathway, *Staphylococcus aureus* infection, and endocytosis (**Figures 5A, B**). GO analysis results demonstrated that changes in the biological processes of DEGs were significantly enriched in the regulation of organelle organization, interstrand cross-link repair, DNA repair, regulation of vascular smooth muscle cell proliferation, vascular smooth muscle cell proliferation, organelle organization, L-amino acid import, regulation of telomere maintenance, telomere organization, and telomere maintenance. Changes in the cellular component of DEGs were mainly enriched in the nuclear DNA–directed RNA polymerase complex, catalytic complex, spindle, RNA polymerase complex, nuclear lumen, nuclear part, histone pre-mRNA 3-end processing complex, mast cell granule, transferase complex, and telomerase holoenzyme complex. Changes in the molecular function of DEGs were significantly enriched in telomerase RNA binding, actin binding, cytokine receptor activity, ubiquitin-like protein transferase activity, ubiquitin-protein transferase activity, peptide antigen binding, endodeoxyribonuclease activity, drug binding, deoxyribonuclease activity, and K63-linked polyubiquitin binding (**Figures 5C–F**).

### Co-Expression Analysis of lncRNA and mRNA

**Figure 6:**
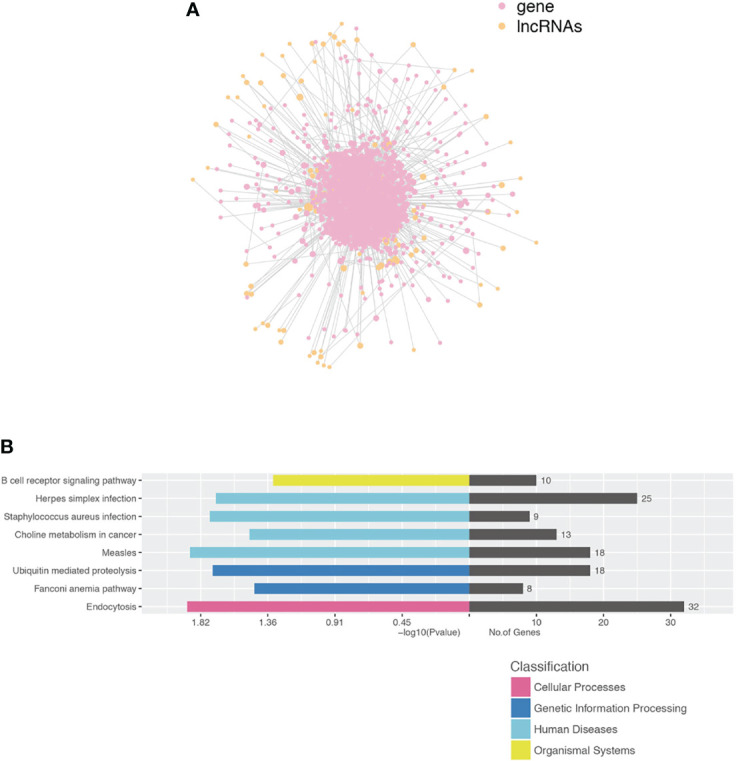
Construction of the lncRNA and protein-coding gene co-expression network. **(A)** LncRNA-TF core network consisting of the top 200 pairs of lncRNAs and TF with the most relevance. TF transcript factor. **(B)** Functional enrichment in each cluster indicated that lncRNAs were functionally associated with mRNAs in particular biological processes.

We performed a co-expression network of lncRNA and mRNA to infer the underlying regulatory function and potential target genes of lncRNA. LncRNAs thart were located within 300K windows downstream or upstream of the given mRNAs were subsequently further studied. Correlated protein-coding genes and lncRNAs were chosen to visualize the correlation of clusters (**Figure 6A**). Furthermore, we analyzed the functional regulatory relationship between the genes. The functional enrichment in each cluster indicated that lncRNAs were functionally associated with mRNAs in particular biological processes, such as the B-cell receptor signaling pathway, herpes simplex infection, *S. aureus* infection, choline metabolism in cancer, measles, ubiquitin-mediated proteolysis, Fanconi anemia pathway, and endocytosis (**Figure 6B**).

### RT-PCR Validation

**Figure 7:**
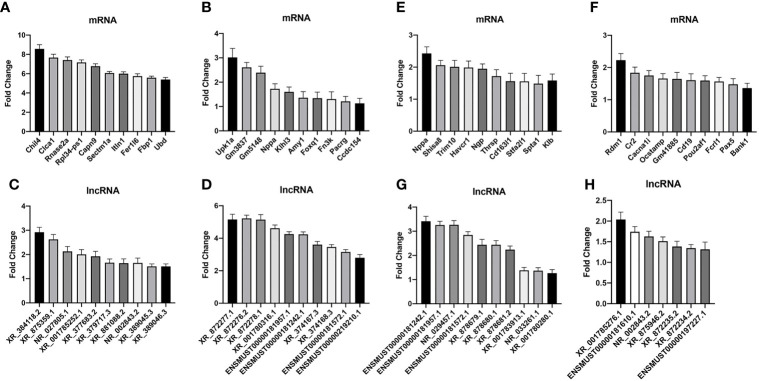
Real-time PCR (RT-PCR) validation of differentially expressed mRNA and lncRNA among the groups. Compared with the control group, differentially expressed mRNAs in WT-PCP mice were shown in **(A)** (upregulated) and **(B)** (downregulated) and differentially expressed lncRNAs were shown in **(C)** (upregulated) and **(D)** (downregulated). In comparison with WT-PCP mice, differentially expressed mRNAs in BAFF-R^–/–^ PCP mice were demonstrated in **(E)** (upregulated) and **(F)** (downregulated) and differentially expressed lncRNAs in **(G)** (upregulated) and **(H)** (downregulated). All *P* < 0.05.

To validate the credibility of the results of microarray analysis, we performed an RT-PCR of the top 10 DEGs. Total RNAs from the lungs of uninfected, WT-PCP, or BAFF-R^–/–^ PCP mice were extracted at 3 weeks after pneumocystis infection. Validation studies further indicated that there were a majority of differently expressed genes among the control group, WT-PCP group, and BAFF-R^–/–^ PCP group, consistent with the RNA sequencing results. Compared with the control group, differentially expressed mRNAs were shown in **Figure 7A** (upregulated) and **Figure 7B** (downregulated) and differentially expressed lncRNAs were shown in **Figure 7C** (upregulated) and **Figure 7D** (downregulated). Validation experiments were also conducted between WT-PCP mice and BAFF-R^–/–^ PCP mice; differentially expressed mRNAs were demonstrated in **Figure 7E** (upregulated) and **Figure 7F** (downregulated) and differentially expressed lncRNAs in **Figure 7G** (upregulated) and **Figure 7H** (downregulated) (all *P*<0.05).

**Figure 8:**
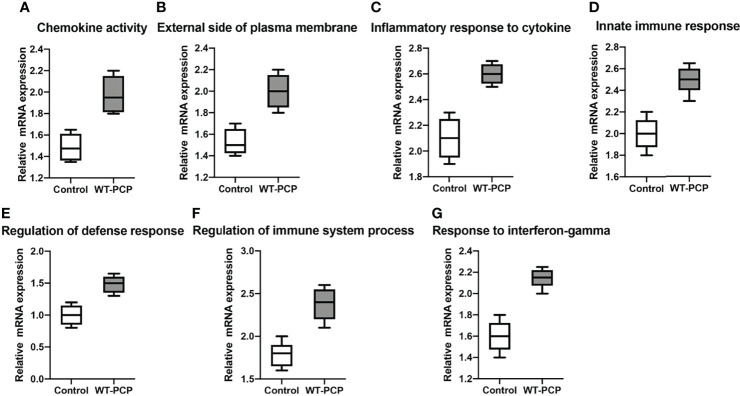
Box-and-whisker plots of the relative mRNA expression values in the WT-PCP mice compared with the uninfected mice. The genes included were related to the respective pathways, including the chemokine activity **(A)**, external side of plasma membrane **(B)**, inflammatory response to cytokine **(C)**, innate immune response **(D)**, regulation of defense response **(E)**, regulation of immune system process **(F)**, and response to interferon-gamma **(G)**. All *P* < 0.05.

**Figure 9:**
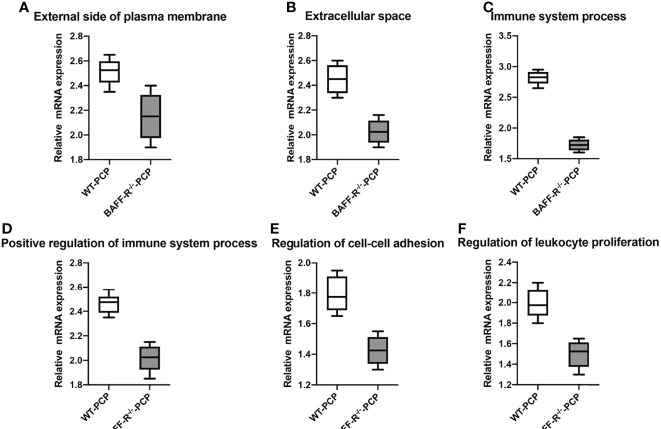
Box-and-whisker plots of the relative mRNA expression values in the BAFF-R^–/–^ PCP mice compared with the WT-PCP mice. The genes included were related to the respective pathways including the external side of plasma membrane **(A)**, extracellular space **(B)**, immune system process **(C)**, a positive regulation of the immune system process **(D)**, regulation of cell–cell adhesion **(E)**, and regulation of leukocyte proliferation **(F)**. All *P *< 0.05.

In addition, according to the results of functional annotation, there was a prevailing elevation of the genes associated with immunity-related pathways in WT-PCP mice compared to the uninfected mice. To validate these results, RT-PCR was conducted on the chemokine activity, external side of plasma membrane, inflammatory response to cytokine, innate immune response, regulation of defense response, regulation of immune system process, and response to interferon-gamma (*P*<0.05, **Figure 8**). We also found that in BAFF-R^–/–^ PCP mice, there was a significant reduction of different gene pathways such as the external side of the plasma membrane, extracellular space, immune system process, positive regulation of the immune system process, regulation of cell–cell adhesion, and regulation of leukocyte proliferation. The results of RT-PCR confirmed the data from microarray analysis as a whole (*P*<0.05, **Figure 9**) (12).

## Discussion

*Pneumocystis* pneumonia (PCP) is an opportunistic, continuously evolving disease challenging clinicians who have a life-threatening illness that inhibits their immune systems (13). PCP was originally described in orphanages after World War II and then gradually observed in acquired immunodeficiency syndrome (AIDS) patients; however, because of the new antiretroviral medications recently approved in HIV-positive patients, the prevalence of pneumocystis has declined in these individuals (14). Accumulating evidence revealed that the increasing use of immunosuppressive agents in hematological malignancy, autoimmune conditions, and transplant recipients has resulted in an increase in PCP in HIV-negative populations (13).

BAFF-R is early expressed in circulating B cells and plays an important role in the maturation of B cells (15). Functional B-cell numbers are significantly decreased in BAFF-R^–/–^ mice. B cells have been demonstrated to play an important role in promoting the proliferation and activation of CD4^+^ T cells and product antibodies in *Pneumocystis* hosts (16–18). However, it is still unclear that how B cells affect T-cell immune responses in *Pneumocystis*-infected individuals. In the present study, using RNA sequencing, we observed that a PCP infection could promote the upregulation of the pathways of both innate and adaptive immunity and knocking out the B-cell receptor–changed expression of lncRNA and mRNA.

A majority of differentially expressed genes related to immune responses were upregulated after a *Pneumocystis* infection. Chil4 is a chitinase-like protein expressed in supporting cells, which plays a novel role in olfactory epithelium regeneration and cell proliferation *via* interaction with inflammatory responses (19). Clca1 is a member of the Clca family and modulates the epithelial cell chloride current and participates in the pathogenesis of mucus hypersecretory-associated respiratory diseases, including asthma, chronic obstructive pulmonary disease, cystic fibrosis, and pneumonia (20). Ubd is a ubiquitin-like protein modifier that targets proteins for proteasomal degradation, and it is upregulated in response to inflammatory stimuli and may play roles in the innate immune system (21). According to the GO and KEGG pathway analyses, the DEGs were mainly enriched in inflammatory cytokine–related pathways such as the TNF, chemokine, cytokine–cytokine receptor, and interferon-gamma. According to a previous study, non-HIV PCP infected individuals were mainly treated by immunosuppressed agents and presented a decreased level of CD4^+^ T cells. However, the immune function of the other immune cells such as NK cells, macrophage cells, and B cells still remains unclear. The results of KEGG analysis revealed that the invasion of pneumocystis could trigger a series of immune responses both related to innate immunity and adaptive immunity. Thus, it is essential to expand the blueprint of various immune cells during a PCP infection to further elucidate the pathogenesis of PCP in a non-HIV population.

The downregulation of genes related to innate and adaptive immunity was confirmed in the BAFF-R^–/–^ PCP mice. Trim10 plays a critical role in the globin gene transcription, and its upregulation is a key factor required for terminal erythroid cell differentiation (12). Ocstamp is essential in bone resorption, and its partners CD9 could promote periodontal bone destruction by the upregulation of Oncogenesis (22). Pou2af1, which regulates B-cell homeostasis and controls humoral immunity, is proved to enhance IL-17A expression in Th17 cells *via* RORgamat (23). Pax5 target genes are mostly expressed in leukemic cells and have been demonstrated as a tumor suppressor in lymphoblastic leukemia (24).. Furthermore, the data of the pathway analysis in this study indicated that BAFF-R deficiency could play a role in the functional regulation of various immunity pathways, such as the chemokine signaling pathway, NF-kappa B signaling pathway, and cytokine–cytokine receptor interaction. This indicated that BAFF-R deficiency could not only have an effect on the cytokine activity and chemokines receptor binding but also influence the cell surface, external side of the plasma membrane, and extracellular space.

In addition, we performed the gene-functional pathway analysis of lncRNA-targeted mRNA, which demonstrated that the pathway was mainly enriched in immunity, metabolism, and the signaling pathway. The results showed that differentially expressed mRNAs were mainly involved in the molecular function terms of the regulation of organelle organization, telomere organization, and telomere maintenance and the biological process terms of cytokine receptor activity and peptide antigen binding. According to these data, we found that the deficiency of BAFF-R could have a significant influence on the immune microenvironment; thus, it might lead to the immunosuppressive patients’ susceptibility to PCP. Non-HIV PCP patients demonstrated the worsen disease progression; however, the treatment of non-HIV PCP has almost only concentrated on TMP-SMZ (trimethoprim–sulfamethoxazole) and caspofungin (25). The differentially expressed pathways in the present study might provide the new target spot of the immune therapy of non-HIV PCP populations.

To investigate the potential regulatory mechanism of the aberrantly expressed lncRNA, the lncRNA, and mrna, the co-expression network of mRNA and lncRNA was built according to the correlation analysis. The data demonstrated that in the core network of lncRNA-TF pairs, the lncRNA could be classified into the categories of pathways such as herpes simplex infection, *S. aureus* infection, and endocytosis, which indicated that the function of transregulatory lncRNA was centralized on immune and infection pathways.

To the best of our knowledge, there are a few studies using microarray technology to reveal the immune regulatory function of cells in PCP. Advances in the genetic characterization of pneumocystis indicates that specific polymorphisms are related to the epidemiology of this pathogen, containing the geographic distribution, medicine resistance, toxicity of several genotypes, and genetics of this population (4). To clarify the characteristics of the genetic diversity of PCP, Magdalena et al. used the sputum specimens of patients with PCP, and the results of the study showed that specific genes such as mtLSUrRNA, CYB, DHPS, and SOD were correlated with certain patient characteristics (18). Using RNA sequencing, some scholars found that Arp9, Sp, and Gsc1 were differentially regulated in the predominant life forms in the PCP population, providing a preliminary annotation of the *Pneumocystis* murina genome (26). Also, a study focused on the antifungal agents of PCP individuals and noted that there were 80 genes up- or downregulated among uninfected, untreated mice and mice treated with anidulafungin to study the metabolism of persisting forms (27).

In summary, this study focused on the non-coding and coding genes in the PCP population. Firstly, the gene sequencing results was compared between the uninfected mice and the PCP- infected mice. The data of DEGs and gene-functional analysis showed that the PCP infection had an effect on the function of various immune cells related to innate immunity and adaptive immunity, which preliminarily revealed the pathogenesis of PCP in non-HIV individuals. Previous studies have verified the central role of CD4^+^ T cells during a PCP infection. In the present study, using BAFF-R^–/–^ mice, we further explored the role of mature B cells in the pathogenesis and disease progression of PCP. The results demonstrated that BAFF-R deficiency plays an important role in the regulation of immune regulatory–related genes and pathways, which indicated that B cells could affect the immune microenvironment in a PCP-infected population. Co-expression network analysis was also performed, and the data showed that there was a variety of differently expressed lncRNAs between uninfected and PCP infected mice. Thus, lncRNA might be provided as novel diagnostic biomarkers and therapeutic targets of PCP. However, in this study, we have not performed further gene-functional experiments. In the future, the gene-functional study of lncRNAs *in vivo* and *in vitro* are required and the blood and BALF samples from patients with PCP are essential for deliberate study.

## Data Availability Statement

The original contributions presented in the study are publicly available. This data can be found here: PRJNA819205.

## Ethics Statement

The animal study was reviewed and approved by Beijing Chao-Yang Hospital, Capital Medical University. No. AEEI-2020-121.

## Author Contributions

Z-HT conceived and designed the research. H-MR and TL performed experiments. H-MR and CZ analyzed data. G-SR, DW, and X-JQ interpreted the results of experiments. CZ prepared figures. H-MR drafted the manuscript. All of the above authors approved the final version of manuscript.

## Funding

This work was funded by the National Natural Science Foundation of China (No. 81870004, No. 81900003), Beijing Natural Science Foundation (KZ201910025031) and Beijing Municipal Administration of Hospital (No. DFL20150302).

## Conflict of Interest

The authors declare that the research was conducted in the absence of any commercial or financial relationships that could be construed as a potential conflict of interest.

## Publisher’s Note

All claims expressed in this article are solely those of the authors and do not necessarily represent those of their affiliated organizations, or those of the publisher, the editors and the reviewers. Any product that may be evaluated in this article, or claim that may be made by its manufacturer, is not guaranteed or endorsed by the publisher.

## Supplementary Material

Supplementary Figure 1 The *Pneumocystis* lung burden was detected by quantitative PCR in WT mice after infection at weeks 1–7.

Supplementary Figure 2 The sequencing data of the transcriptomics analysis of lung tissue. **(A)** Saturation of all the genes were detected. **(B)** The RPKM density distribution map of genes. **(C)** The results of PCA analysis of samples.

Supplementary Figure 3 Methodology workflow.
