# Supplementary figures and images for: Analysis of lncRNA and mRNA Repertoires in Lung From BAFF-R-Deficient Pneumocystis-Infected Mice
Source: Front Immunol. 2022 Jun 10;13:898660. doi: 10.3389/fimmu.2022.898660 (PMC9238325; doi:10.3389/fimmu.2022.898660)

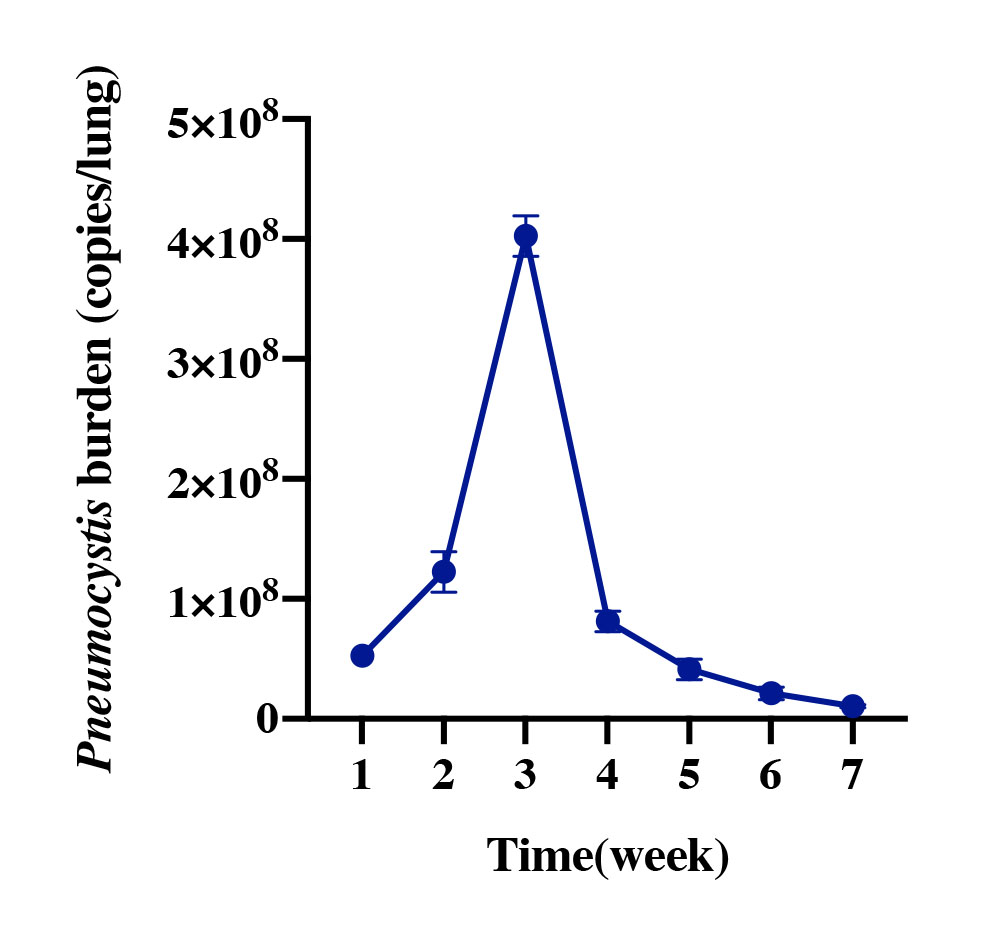

Supplement: Supplementary Figure 1 — The Pneumocystis lung burden was detected by quantitative PCR in WT mice after infection at weeks 1–7. [file Image_1.jpeg]

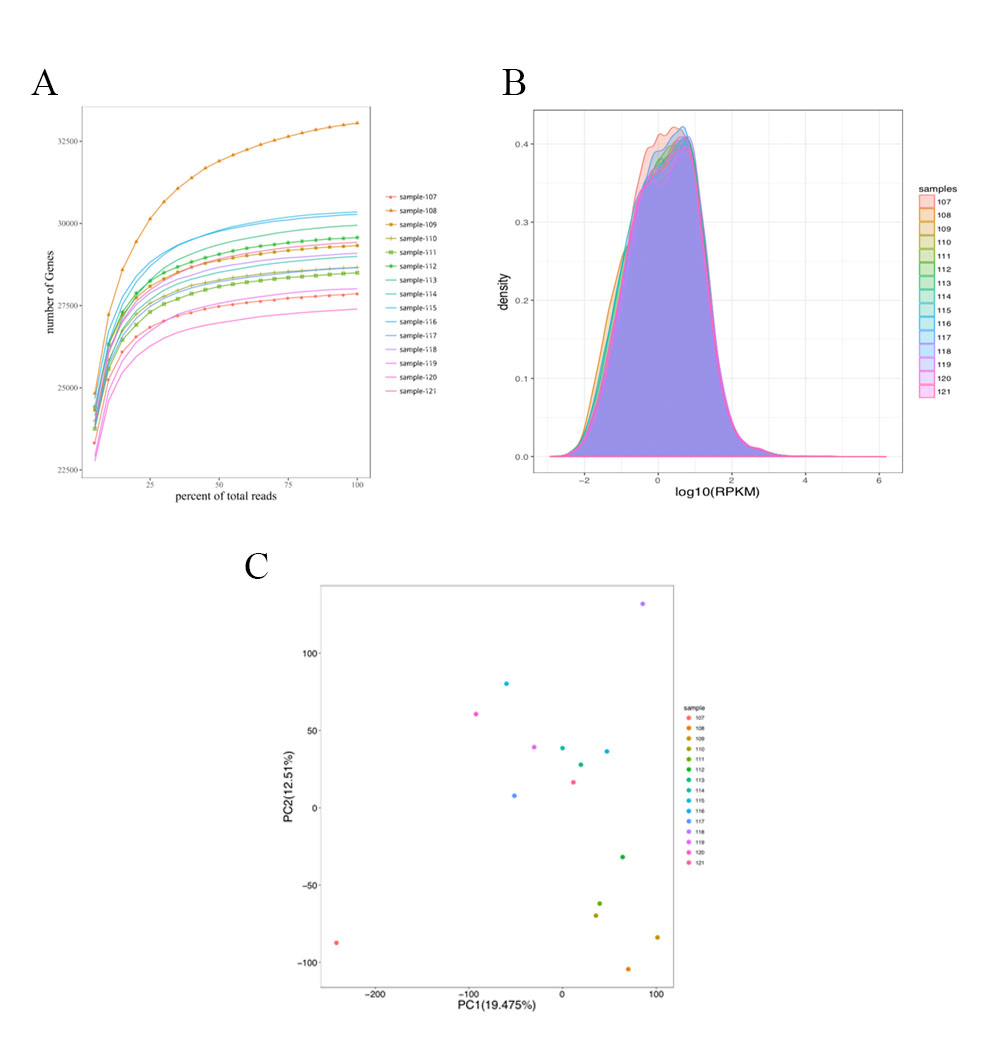

Supplement: Supplementary Figure 2 — The sequencing data of the transcriptomics analysis of lung tissue. (A) Saturation of all the genes were detected. (B) The RPKM density distribution map of genes. (C) The results of PCA analysis of samples. [file Image_2.jpeg]

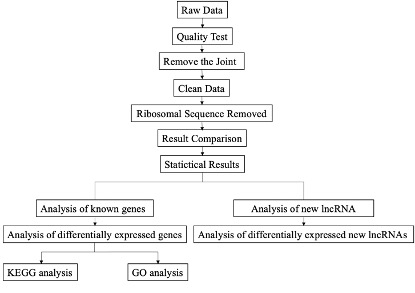

Supplement: Supplementary Figure 3 — Methodology workflow. [file Image_3.jpg]
